# Teaching Kitchens and Culinary Gardens as Integral Components of Healthcare Facilities Providing Whole Person Care: A Commentary

**DOI:** 10.3390/nu15194162

**Published:** 2023-09-27

**Authors:** Angela M. Fals, Andrea M. Brennan

**Affiliations:** 1 AdventHealth for Children, 601 E. Rollins St., Orlando, FL 32803, USA; 2 AdventHealth Research Institute, 800 N. Magnolia Ave., Orlando, FL 32803, USA; andrea.brennan@adventhealth.com

**Keywords:** culinary medicine, teaching kitchen and garden, healthcare system, pediatric obesity, employee wellness

## Abstract

Child and adult obesity continue to be major health concerns in the United States and can contribute to the development of chronic diseases. Culinary medicine, which incorporates teaching kitchens and gardens, may be a useful strategy for preventing and/or treating obesity-related disease by providing the knowledge and skills that encourage consumption of whole plant-based foods prepared at home. Though emerging research describes the benefits of culinary medicine-based programming, examples of teaching kitchens and culinary gardens being integrated into current clinical practice is minimal. Here, we describe the development of innovative, community-centered culinary medicine programming borne from interdisciplinary collaboration at a leading healthcare system. Preliminary outcomes suggest improvements in anthropometrics, cardiometabolic risk factors, and participation in healthy lifestyle behaviors in pediatric weight management patients, as well as improved confidence, knowledge, and likelihood to prepare whole food, plant-based meals in healthcare employees following participation in culinary medicine workshops. Hospitals and culinary medicine partners can support each other through shared knowledge, vision, and resources to provide value-based care to patients in the community. Collaboration among gardeners, chefs, architects, educators, and healthcare professionals can transfer traditional physician-driven care to patients, empowering them with the tools, resources, and confidence to improve health and wellbeing.

## 1. Introduction

Childhood and adult obesity continue to be major health concerns in the United States. According to the 2017–2020 National Health and Nutrition Examination Survey (NHANES), one in six (16.1%) children and adolescents aged 2–19 years were overweight and one in five (19.7%) were in the obesity category. Higher prevalence rates were observed among Hispanic and non-Hispanic black children compared to non-Hispanic white and Asian children [1]. Severe obesity is the fastest growing subcategory of obesity in American adolescents and is associated with medical complications and comorbidities which can advance into adulthood, including increased cardiometabolic risk, breathing problems, and orthopedic concerns [1]. Similarly, the obesity prevalence in adults is alarming. In 2017–2018, 30.7% of adults in the United States were in the overweight category, 42.4% had obesity, and 9.2% had severe obesity [2]. Many obesity-related chronic diseases are lifestyle-based, preventable, and even reversible, especially in children and adolescents [3].

Culinary medicine, which includes the use of teaching kitchens, is an evidence-based approach to nutrition education, which blends the art of cooking with the science of medicine [4]. Teaching kitchens provide nutrition-based knowledge and tools to individuals to promote the consumption of whole foods prepared at home [5]. The demonstrated benefits of teaching kitchen classes include food cost savings, increased nutrition and culinary knowledge, and improved confidence in the ability to regularly practice a healthier diet [6,7,8]. Prior studies have shown that teaching kitchen-based interventions lead to increased vegetable consumption and preparation of home-based meals [6] in addition to increased adherence to the Mediterranean diet [8], all of which improve dietary sodium intake and downstream chronic disease prevention [9]. Additionally, Moore et al. measured micronutrient adequacy and diet quality in 38 healthcare and university employees who participated in a 10-week teaching kitchen program that coupled didactic sessions with experiential hands-on cooking [10]. Though not statistically significant, the proportion of participants with high micronutrient adequacy increased from 30% to 48% at 3-month follow-up and the seafood/plant protein score of the Healthy Eating Index significantly increased [10]. While teaching kitchens have received increased attention from the healthcare community over the last several years [5], there are limited examples of multi-disciplinary collaboration involving architects, chefs, and hospital systems in the formation of teaching kitchens on or in close proximity to healthcare campuses [11].

The purpose of this commentary is three-fold: (1) to describe the development of an innovative, community-centered nutrition and culinary medicine education program at a pediatric weight management medical clinic and show preliminary evidence of its impact; (2) to describe other organizational efforts formed by interdisciplinary collaboration of community visionaries and healthcare leaders through a local non-profit teaching kitchen and culinary garden; and (3) to describe the expansion of a culinary medicine program for healthcare employees and demonstrate preliminary evidence of its impact on willingness to participate, confidence, and skills. The intent is to demonstrate how passionate thinkers from different, yet complementary sectors in the health and nutrition space collaborate to ensure the success and longevity of impactful programs, show initial health outcomes for past and current patients, and provide inspiration to other systems and organizations by sharing experiences and lessons learned.

## 2. Development of the Pediatric Weight and Wellness Program

In 2010, AdventHealth for Children (AHFC)/AdventHealth Medical Group (AHMG) along with a board-certified physician specialist in pediatric and obesity medicine who has additional certification in culinary medicine initiated the Pediatric Weight and Wellness (PWW) medical practice and program (Pediatric Weight Loss and Wellness|AdventHealth for Children; accessed on 25 September 2023 https://www.adventhealth.com/hospital/adventhealth-children/pediatric-weight-loss-and-wellness-programs). The aim of the PWW program is to address childhood obesity using a multidisciplinary approach to treatment and prevention of weight-related comorbidities that combines whole person care, including medical, psychological, nutritional, and physical activity. The PWW program accepts children and adolescents aged 5–18 years old with a body mass index (BMI) greater than or equal to 85% (overweight, obesity and severe obesity categories).

It provides the following services:

1. Medical supervision and ongoing assessment of associated weight-related comorbidities with a pediatric obesity medicine specialist.
2. Psychological evaluations and support provided by a child health clinical psychologist addressing issues such as body dysmorphia, motivation, individual and family behavioral changes, and screenings for depression and anxiety.
3. Individualized nutrition plans by a dietitian with certification in pediatric weight management.
4. Exercise education and assessments along with personal and group fitness sessions provided by an exercise physiologist with certification in personal training by the American College of Sports Medicine, in addition to partnership with local fitness facilities within and outside of the hospital system.
5. Parent and child/teen health coaching provided by health and wellness educators.
6. Experiential cooking and garden education classes consisting of age-appropriate health education sessions with an emphasis on treatment or prevention of weight-related comorbidities.
7. Teen and parent support groups
8. Hospital-affiliated resources for at-risk families experiencing food insecurity which became more pronounced during the COVID-19 pandemic (2020–2022).

Since its inception, AHFC has remained committed to the health and wellness of obese/overweight children in the Central Florida community, supporting the full clinical multidisciplinary team and basic program components. Philanthropy remains crucial to the long-term financial sustainability of the extended PWW programming components. Program funding has been provided by over 90 local and national organizations and partnerships, ranging from gifts in-kind, providing incentives/rewards for program participants, to multi-year grant funding from local and national organizations.

### 2.1. PWW Program Culinary Medicine Workshops

As the PWW medical practice and program evolved, it became clear to the medical providers and allied health practitioners that most of the children, teenagers, and families involved in the PWW program did not have the basic skills and understanding to prepare healthy, home-cooked meals. Instilling the confidence and knowledge to identify healthy food, prepare meals at home with fewer processed ingredients, and make healthy choices in restaurants, schools, and social settings became a vital part of the PWW program.

Initially, in partnership with the AdventHealth Diabetes Institute’s teaching kitchen space, the PWW program began facilitating cooking classes with a chef educator and PWW dietitian. Gardening workshops, facilitated by a volunteer from a local garden store who provided instruction time and supplies such as soil, tools and seedlings, took place in the evenings or weekends several times per month in the PWW program office lobby or green space in front of the office building. Concurrently, the volunteer garden educator at Orlando Junior Academy (OJA) was teaching OJA students and teachers to combine hands-on garden education with the elementary curriculum. Over time, the PWW program participants also started attending gardening workshops after hours at the school teaching garden within OJA. A description of the workshops is provided in Table 1.

### 2.2. Partnership with Edible Education Experience at the Emeril Lagasse Foundation Kitchen House and Culinary Garden

**Figure 1:**
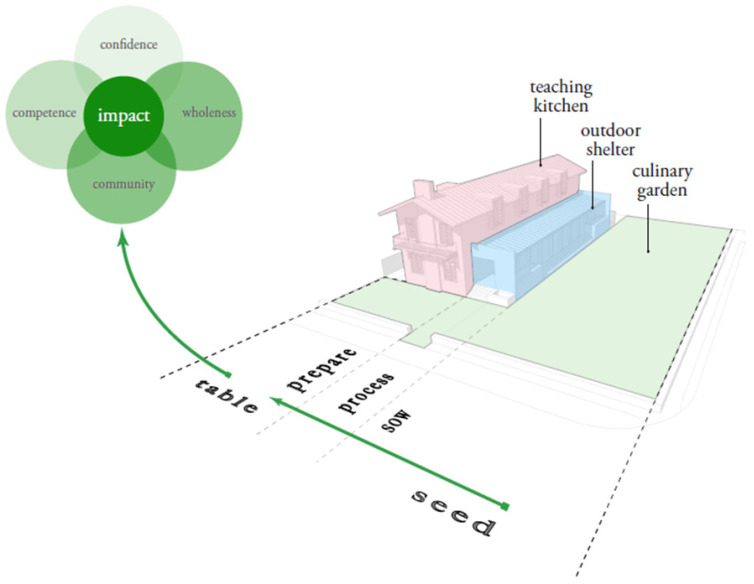
Impact of the seed-to-table continuum. Seed-to-table experiences at Edible Education Experience can have an impact on developing competence, confidence, community, and wholeness.

As AHFC PWW and culinary medicine programs continued to grow, simultaneously, the Edible Education Experience (EEE) in Orlando was in development. A group of community wellness innovators came together from the following backgrounds: medical, gardening, culinary, architecture, and academics. Edible Education Experience is a non-profit organization that became incorporated in 2014 and opened in 2017. It operates the award-winning (American Institute of Architects Orlando Chapter Award of Honor) Emeril Lagasse Foundation Kitchen House and Culinary Garden, a teaching kitchen and on-site culinary garden. It is based on the belief that seed-to-table experiences “forge meaningful relationships, develop autonomy and empowerment, build competency with relevance to the natural world and one’s own lives, encourage a shared sense of responsibility, and enable food to become an artistic tool for shifting perspectives” [12]. Combined with “Food is Medicine” programming, seed-to-table experiences at EEE can have an impact on developing competence, confidence, community, and wholeness, demonstrated in Figure 1. The shared work and dedication to health and wellness of AHFC PWW and EEE has fostered continued growth of nutrition and culinary education in the Central Florida community. The partnership has evolved from providing a location for nutrition and culinary programming, to the shared development of a culinary medicine curriculum.

The successful partnership between the teaching kitchen and culinary garden and the pediatric practice is made possible by the vital role of key health and wellness advocates at AHFC, AH, and EEE. The Culinary Educators coordinate special programming for adults and children/teens, engage community chefs, design employee wellness programming, oversee kitchen volunteers and assistants, and execute corporate team builders. The Garden Educator manages the 3000 square foot culinary garden, including planning, planting, and harvesting the crops; coordinates “edible lessons” with teachers and culinary educators; provides hands-on experiences to visitors; and oversees the garden volunteers and assistants. The Executive Director steers the organization by managing operations, leading business development with help from the Business Coordinator, fundraises, oversees facility rentals, and works with the EEE team, Board of Directors, and community partners. Capital funders for the Emeril Lagasse Foundation Kitchen House and Culinary Garden were: AdventHealth for Children, the Emeril Lagasse Foundation, Orlando Junior Academy, and other forms of philanthropy, like private donations and gifts-in-kind.

**Figure 2:**
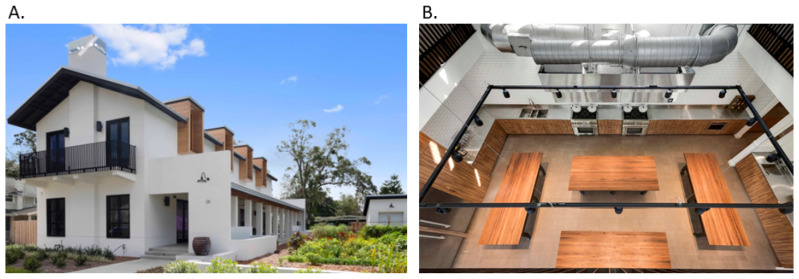
The Emeril Lagasse Foundation Kitchen House and Culinary Garden in Orlando, Florida: (**A**) exterior (veranda) and (**B**) aerial view of interior.

**Figure 3:**
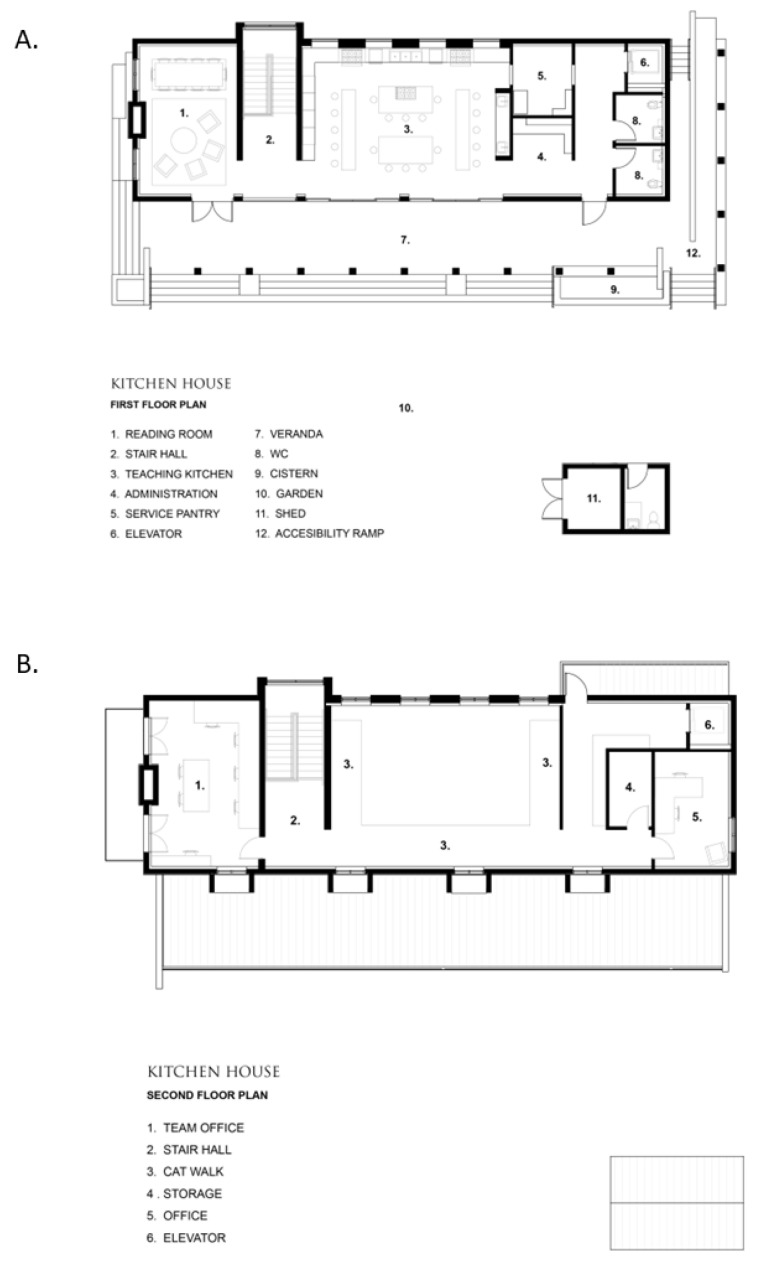
The floor plan of the Emeril Lagasse Foundation Kitchen House and Culinary Garden: (**A**) first floor; (**B**) second floor.

Figure 2 and Figure 3 show the Kitchen House and Culinary Garden indoor and outdoor design and floor plan.

After its grand opening, the EEE Kitchen House and Culinary Garden became the primary site of the gardening and culinary medicine workshops for children and teens of the AdventHealth for Children PWW program. The partnership has evolved from providing the backdrop and location for workshops teaching hands-on nutrition, gardening, and culinary education, to developing a shared curriculum for seed-to-table experiences within the framework of culinary and lifestyle medicine. The partnership between the EEE and the AHFC PWW program is featured in the Teaching Kitchen Collaborative Food is Medicine Map [13].

### 2.3. Preliminary Outcomes of PWW Culinary Medicine Workshops

The PWW team retrospectively analyzed patient outcome data following 6 months of participation in the PWW program from 2022–2023. Participation in the PWW program included behavioral modification interventions/counseling in motivation, nutrition, and exercise; participation in culinary medicine workshops; and clinical interventions, such as screening for and treatment of medical/mental health co-morbidities and, when appropriate, the use of medications (such as metformin for severe insulin resistance or type 2 diabetes mellitus). Patients lost on average 3.67 ± 3.24 kg (range: −0.4 to −13.88 kg) and reduced BMI by 1.29 ± 0.93 kg/m^2^ (range: −0.10 to −3.70 kg/m^2^).

Changes in anthropometric measurements, cardiometabolic risk factors, and surveyed lifestyle behaviors over the 6-month timeframe are shown in Table 2. Most pediatric patients showed notable improvements or maintenance in each of these categories. For anthropometrics, they showed improved or maintained body fat percentage (65.0% of participants), waist (55.1% of participants), and hip (63.6% of participants) measurements. Clinically, we observed a decreased or maintained risk of prediabetes and diabetes demonstrated by improved and/or maintained high-fasting blood glucose ≥ 100 mg/dL (78.3% of participants), high-fasting insulin ≥ 20 mIU/L (74.4% of participants), and high hemoglobin A1C ≥ 5.7% (77.4% of participants) within the normal range. For cardiovascular disease risk factors, most patients improved and/or maintained high systolic blood pressure ≥ 95th percentile (100.0% of participants), diastolic blood pressure ≥ 95th percentile (77.8% of participants), total cholesterol ≥ 170 mg/dL (77.8% of participants), LDL-cholesterol ≥ 110 mg/dL (80.0% of participants), and triglycerides ≥ 100 mg/dL for children under 10 years and ≥130 mg/dL for children over 11 years (71.9% of participants) within the normal ranges. Indeed, the number of participants who improved in two or more cardiometabolic risk factors was 76.0%. Similarly, most participants improved and/or maintained all surveyed lifestyle behaviors (59.1–84.4% of participants) including nutrition, physical activity, and quality of life. Lack of transportation, difficulty with scheduling, and issues with obtaining blood draws contributed to the lower sample size observed for laboratory measures.

**Figure 4:**
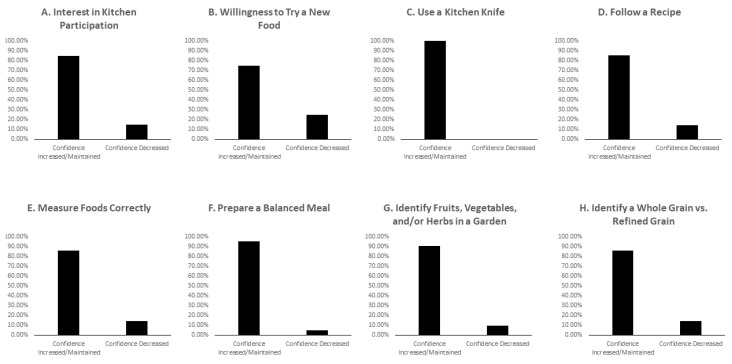
Proportion of pediatric patients whose perceived confidence in their skills and knowledge were maintained/improved or decreased following a series of four culinary medicine workshops.

Figure 4 demonstrates pediatric patients’ perceived skills and knowledge change before and after attending a series of four culinary medicine workshops. Patients’ interest in participating in the kitchen (85.0% of participants) and their willingness to try a new food (75.0% of participants) were improved or maintained following the workshops. Patients also improved or maintained their confidence using a kitchen knife (100.0% of participants); following a recipe (85.7% of participants); measuring foods correctly (85.7% of participants); identifying fruits, vegetables and/or herbs in a garden (90.5% of participants); identifying a whole grain versus a refined grain (85.7% of participants); and preparing a balanced meal (95.2% of participants).

These findings suggest promise for the PWW program and, specifically, the culinary medicine workshops, in improving multiple facets of behavioral and physical health, including biomarkers in pediatric patients, when combining clinical expertise with culinary medicine education at teaching kitchens and gardens. Formal research studies are warranted to determine the program’s efficacy and effectiveness more robustly, in addition to the effect of the culinary medicine components, per se, independent of a clinical pediatric obesity program.

## 3. Organizational Efforts to Extend the Influence of Teaching Kitchens and Culinary Gardens within the Context of Culinary Medicine

### 3.1. Development of Advisory and Governance Boards

In 2019, the AdventHealth Health and Wellness Advisory Board and the AdventHealth Culinary Medicine Governance Board were established. These boards have representation from multiple disciplines and departments including high level administration, medical providers, population health, media relations, consumer marketing, and employee health and wellbeing.

The shared aims of these boards are to help align nutrition education programming across the AdventHealth Central Florida system and beyond; clearly project the organization’s stance on health and wellness to the community; streamline resources and maximize program reach; and attract local and national partners for long-term financial support and program sustainability. Whole person health with a focus on healthy lifestyles over a lifetime is a major goal at AdventHealth. These boards will continue to provide a vehicle for successful collaboration between AdventHealth departments and facilitate the development of appropriate, evidence-based messaging in health and wellness to the community.

### 3.2. Culinary Medicine Curriculum Guide

The AdventHealth Culinary Medicine Governance Board curriculum subcommittee developed a major advance in culinary medicine programming, the “AdventHealth Culinary Medicine Curriculum Guide”. This guide provides clarity on the vision, goals, and philosophical approach for nutrition and cooking education classes for the intended audiences of the AdventHealth Central Florida community. Both general “back-to-basics” cooking and gardening classes as well as disease-specific classes with emphasis on chronic disease prevention and treatment are included. The guide also includes extensive reviews of evidence-based support for curricula, overarching and guiding principles, the culinary medicine curricula consensus, and asset mapping that includes partner gardens, farms, teaching kitchens, and a recipe database.

### 3.3. Books, Film, and Resource Development

Continued multi-departmental collaboration between the participants on the AdventHealth Health and Wellness Advisory Board and the Culinary Medicine Governance Board, in conjunction with AdventHealth Press, developed several community resources.

During the COVID-19 pandemic, a digital cookbook entitled “Plant-Powered Recipes to Help You Feel Whole”, was created and provided to the hospital community and patients free of charge [14]. The plant-based recipes and educational articles from nutrition and lifestyle medicine experts were designed within the context of whole person care to help patients and their families feel in control of certain aspects of their health during a time of apparent chaos resulting from the pandemic.

A series of books was also developed under the title “Eat Plants Feel Whole”. This includes an educational trade book with 50 plant-based recipes and an accompanying journal to personalize the patient health and wellness journey [15]. The third book in the series, “Eat Plants Feel Whole Cookbook: Prevent Disease, Restore Your Health, Energize Your Life” has been developed with an emphasis on children, teens, and families and is slated for publication soon. These resources continue to promote consumption of whole, plant-based foods and participation in healthy lifestyles for families featuring over 160 original recipes combined.

The documentary film entitled PlantWise (available worldwide for free with 18 subtitled languages at www.PlantWiseFilm.com; accessed on 25 September 2023) was developed to inform viewers about the transformative, life-changing power of a plant-based diet while following six patients with chronic diseases on their journeys towards renewed vitality. Included is commentary from 18 lifestyle medicine experts from 14 medical specialties. It is being utilized by hospitals and healthcare systems, companies for employee wellness, medical schools for physician and allied health training, and physician private practices.

The cookbook ”Simply Healthy: The Art of Eating Well, Diabetes Edition” with over 70 recipes with a “plant-slant” focus was developed specifically for patients with prediabetes or type 2 diabetes [16] by AH Diabetes Institute and is being incorporated into the prediabetes/diabetes-specific PWW Culinary Medicine workshops so that every qualifying patient in PWW receives a copy for their family.

Most recently, the book “50 Questions & Answers on Family Nutrition and Wellness” was authored by a pediatrician specializing in obesity and culinary medicine, a child clinical health psychologist, three registered dietitians, and an exercise physiologist. It was designed specifically for parents and answers the most common questions child health experts receive. A free, electronic version of the book will be shared with patients’ parents at AHFC and AH.

Finally, a recipe database was created to house 250 recipes developed for the projects previously listed with potential for future growth. It is the goal that these valuable resources are made available for multiple uses, including culinary and lifestyle medicine, patient education, employee nutritional services, health fairs, newsletters, and online patient portals.

### 3.4. Employee Wellness

Due to continued interdepartmental collaboration, in 2020 the partnership with EEE expanded to include programming for AdventHealth employees under the Employee Health and Wellbeing team. Together, the groups developed a curriculum for seed-to-table experiences within the framework of culinary and lifestyle medicine. AdventHealth employees were offered a three-class series as part of the wellness incentive program, in which participants gain points for completing biometric, educational, and goal-setting activities. If employees accumulate enough points, they receive an end-of year financial bonus. The classes were adapted from the American College of Lifestyle Medicine culinary medicine curriculum for health professional training program [17]. The series included a sensory garden tour with emphasis on seasonal crops, three cooking classes with the EEE team, and a question-and-answer session with an AdventHealth dietitian. Participants also receive a digital booklet of the co-branded (AdventHealth and EEE) “Recipes, Nutritional Facts and Health Benefits” that includes recipes, ingredients, and descriptions of the associated health benefits of the seed-to-table experiences.

During the COVID-19 pandemic, the employee series pivoted to virtual classes, including a real-time garden tour in 2020–2022. The Kitchen House and Culinary Garden was equipped to offer virtual educational programming, and as pandemic restrictions lifted it allowed a safe return to face-to-face programming with an appropriate, well-ventilated kitchen space for social distancing and the outdoor garden area. Due to the success of the virtual option, current programming at EEE for AH employees is now a hybrid of three engaging virtual sessions and one in-person class focused on building confidence, skills, and community.

Session 1 includes an introduction to seed-to-table and whole food, plant-based eating. Lead culinary and garden educators discuss food values (i.e., cost, taste, nutrition, sustainability, grown locally, calorie density, seasonality, social/environmental/mental impacts). The difference between processed and unprocessed whole foods is examined and nutritional connections are drawn between eating different parts of a plant (i.e., stem, leaf, root, flower). Participants step virtually into the culinary garden, learning about the journey food takes from seed to table and several whole food, plant-based recipes are made together.

Session 2 discusses food groups and flavor techniques. The lead garden educator discusses which foods are growing in the current season and introduces composting, drawing parallels between the microorganisms of a garden to that of the human body. For the culinary portion of this session, the focus is on knife skills for seasonal root vegetables and cooking techniques (e.g., steaming vs. roasting). Educators encourage participants to taste and adjust flavors as needed when cooking to build autonomy in the kitchen.

Session 3 discusses sugar science and desserts. Participants are introduced to forms of sugar found naturally in foods and discuss what sugar-added foods are. The impact of different cooking or preparation techniques and how they interact with sugar is demonstrated, such as caramelizing vegetables and freezing bananas. Participants also openly discuss food choices and preferences. In this session, the beauty of food is celebrated, taking time to plate dishes and practice awareness through an observation-based mindful eating activity.

The in-person session provides a space for participants to connect with one another while preparing a Mediterranean-style meal together. Participants practice knife skills that were previously described in virtual sessions, recognize flavor additives and techniques to enhance flavor, follow recipes that use general cooking and preparation techniques, and identify how values influence daily choices around food and health.

Plans to expand opportunities for physicians, residents and medical students are currently underway.

#### Employee Health and Wellbeing Program Preliminary Outcomes

Employees who participated in EEE programming between 2021 and 2022 were surveyed before and after completing the sessions described above. One hundred and sixty-two employees completed the survey prior to entering the program and 28–52 employees completed the survey after the program ended, varying by survey. Changes in confidence, likelihood, and influences of preparing whole food, plant-based meals are shown in Table 3.

The percentage of participants who felt somewhat and/or very confident in his or her ability to design and prepare a balanced plant-based meal, choose nutritious foods, identify the difference between unprocessed and processed food, identify foods growing in the garden, incorporate seasonal foods into meals, and identify a whole grain and refined grain increased following participation in the employee series. Additionally, the number of participants who felt confident using a kitchen knife and other tools while preparing a meal also increased.

The percentage of participants who were likely to prepare a new recipe, eat plant-based meals cooked at home, engage in conversations about food choices, and share cooking skills or knowledge increased following program participation. Furthermore, the percentage of participants who placed high importance on cost, nutritional value, taste, environmental impact, social impact, locally grown produce, calorie density, and seasonality for influencing food choices also increased.

Employee participation in culinary medicine programming improved confidence and likelihood for healthy meal preparation. These findings should be systematically assessed with validated surveys and scientifically tested in future research to confirm the role of teaching kitchens and culinary gardens in improving employee health and wellness.

## 4. Lessons Learned and Opportunities

Value-based care is increasingly becoming a central tenet of healthcare systems in the United States. The partnership between the AH and EEE teams at the Kitchen House and Culinary Garden to form programming integrated into the pediatric and employee wellness practice at AdventHealth may provide promising lessons for extension to the hospital system at large. The successful collaboration between hospital systems and teaching kitchens and culinary gardens can add value for patients within their community. There are several ways that hospitals and teaching kitchen programs can mutually support each other to provide sustainable programming that meets each organization’s community needs.

Hospital systems can support teaching kitchens and culinary gardens by:

1. Providing access to experts in various health-related fields, including medicine (primary and specialty care physicians and providers), psychologists, dietitians, and educators. Experiential seed-to-table experiences in combination with a didactical component in culinary medicine are effective in actively engaging participants [5].
2. Providing expertise in data acquisition, outcome development, and research methods for demonstrating the efficacy and effectiveness of their program to the scientific community.
3. Providing teaching models for healthy lifestyles.
4. Providing medical leadership for the collaborative development of an evidence-based culinary medicine curriculum.
5. Providing financial resources, such as working with hospital foundations or assisting with obtaining community grants.
6. Providing support through the organization’s governance or advisory boards.

Teaching kitchens and culinary gardens can support hospital systems by:

1. Providing research and learning “laboratories” as locations for the early determination of effective interventions in culinary medicine [5].
2. Providing access to culinary experts including garden and culinary educators, chefs, gardeners, and farmers.
3. Providing effective seed-to-table teaching models for culinary and garden education while empowering the community to make healthy food choices.
4. Providing culinary and garden leadership and expertise in the collaborative development of an evidence-based culinary medicine curriculum.
5. Providing a platform for increased community engagement.
6. Providing program flexibility, including a safe outdoor meeting space, well-ventilated indoor kitchen, and virtual programming in times of public health crises such as the recent COVID-19 pandemic.

## 5. Conclusions and Future Directions

The recent White House National Strategy on Hunger, Nutrition, and Health, released in 2022, demonstrates federal support for ending hunger, promoting health, and reducing diet-related diseases in Americans [18]. This national strategy emphasizes five pillars, including: (1) Improve food access and affordability; (2) Integrate nutrition and health; (3) Empower all consumers to make and have access to healthy choices; (4) Support physical activity for all; and (5) Enhance nutrition and food security research. The adoption of culinary medicine programs into health care settings that include teaching kitchens and culinary gardens aligns with several of these pillars and should be researched further and considered more widely.

Culinary medicine, including teaching kitchens and culinary gardens, is an emerging field with the potential for changing how healthcare systems interact with their communities. Organizations should be encouraged to integrate culinary medicine into clinical practice, particularly in areas such as pediatric obesity management, wherein learning culinary skills at a young age may promote access to and knowledge of healthy eating behaviors that will continue into adulthood. Future priorities include expanding culinary medicine programming within the context of whole person care beyond pediatric obesity medicine; engaging physicians/medical providers, and trainees through education opportunities; investigating the feasibility of incorporating the teaching kitchen and culinary garden into shared medical appointments (in-person or virtual); and formally evaluating the efficacy and effectiveness of the programs for pediatric obesity prevention and treatment as well as employee wellness.

Teaching kitchens and culinary gardens extend the culture of health and wellness beyond the hospital and clinical walls in which food is used to heal and restore wellbeing. Collaboration among gardeners, chefs, architects, educators, and healthcare professionals can transfer traditional physician-driven care to patients, empowering and equipping them with the tools, knowledge, resources, and confidence to actively promote health and wellbeing for themselves. Hospital systems should consider integrating culinary medicine programming into their practice by forging community partnerships that include teaching kitchens and culinary gardens integrated as “shared assets” into their own campuses [5]. Implementing non-traditional care strategies can lead to cutting-edge changes in healthcare delivery.

## Figures and Tables

**Table 1:** Pediatric Weight and Wellness Program culinary medicine workshop descriptions.

Workshop	Education Topics Covered	Hands-On Experiences	Disease-Specific Topics
Culinary Garden	Food groups and the balanced plate Food safety and kitchen skills Meal planning and meal preparation	Garden Tour: Gardener teaches participants about herbs, spices, and how to identify plants in the garden Prepare “meal-to-go box” Culinary skills training	Prediabetes/Diabetes *: meal planning and preparation; using herbs for flavor; low glycemic choices and whole foods; portion control CVD **: Using herbs to flavor; reducing sodium content; lean protein alternatives; reducing saturated fat; increasing fiber intake
Garden to Table	Food systems, seasons and seed lifecycle	Gardening Composting Mindful tasting Measuring ingredients Kitchen safety Basic knife skills	Prediabetes/Diabetes: reviewing food labels; portion control and mindful eating; low glycemic foods; reducing added sugars CVD: Reducing saturated fat; increasing fiber; reducing added sugar intake
Kitchen Creations	Portion control Culinary skills	Prepare a full meal Learn to peel, chop, measure, mix, cook, and roast	Prediabetes/Diabetes: portion control and measurement; reducing added sugars and glycemic load; incorporating whole grain foods CVD: Lean protein alternatives; reducing sodium intake; increasing fiber intake
Culinary Medicine Prediabetes/Type 2 Diabetes	Carbohydrate counting Meal planning and timing Carbohydrate sources Glycemic index	Identify added sugars	Prediabetes/Diabetes: Basic understanding of how to optimize blood sugar control by meal timing and planning CVD: reducing saturated fat and sodium intake; increasing fiber intake; building flavor with herbs vs. seasoning
Grocery Store Tour and Scavenger Hunt	Reading food labels	Evaluate nutrition facts labels Identify high risk processed foods that contain more sodium, added sugar, and calories Find replacement options within varied budgets	Prediabetes/Diabetes: Identifying food with lower glycemic load CVD: Identifying foods with higher sodium and saturated fat and seeking alternatives
Know Your Body	Anatomy and physiology of the human body Healthy behaviors	Movement and stretching	Weight-related comorbidities including fatty liver disease, CVD, prediabetes/type 2 diabetes, asthma, gastroesophageal reflux, cancer

* Prediabetes/Type 2 Diabetes Mellitus; ** CVD, cardiovascular disease.

**Table 2:** Changes in health outcomes following 6 months of participation in the Pediatric Weight and Wellness Program in 2022–2023.

Targeted Metrics	*n*	Improved and/or Maintained (*n*, %)	Worsened and/or Declined (*n*, %)
Anthropometrics			
% Body Fat	100	65 (65.0%)	35 (35.0%)
Waist *	143	59 (55.1%)	48 (44.9%)
Hip *	143	68 (63.6%)	39 (36.5%)
Cardiometabolic Risk Factors			
Total Cardiometabolic Risk (Improved ≥2 risk factors)	25	19 (76.0%)	6 (24.0%)
High Blood Pressure (Systolic ≥ 95%)	9	9 (100.0%)	0 (0%)
High Blood Pressure (Diastolic ≥ 95%)	9	7 (77.8%)	2 (22.2%)
High Insulin (≥20 mIU/L)	78	58 (74.4%)	20 (25.6%)
High Blood Glucose (≥100 mg/dL)	23	18 (78.3%)	5 (21.7%)
High HbA1c (≥5.7%)	31	24 (77.4%)	7 (22.6%)
High LDL Cholesterol (≥110 mg/dL)	45	33 (73.3%)	12 (26.7%)
High Total Cholesterol (≥170 mg/dL)	48	34 (70.8%)	14 (29.2%)
High Triglyceride (≥100 mg/dL 10 years and under/≥130 mg/dL 11 years and over)	62	48 (77.4%)	14 (22.6%)
Low HDL Cholesterol (≤40 mg/dL)	32	21 (65.6%)	11 (34.4%)
Quality of Life			
Elevated PSC (≥28) **	16	12 (75.0%)	4 (25.0%)
Sleep and Activity			
Screen Time	101	83 (82.2%)	18 (17.8%)
Sleep	232	152 (65.5%)	80 (34.5%)
Family Activity	198	140 (70.7%)	58 (29.3%)
Individual Activity	198	117 (59.1%)	81 (40.9%)
Nutritional Intake			
Sugar Sweetened Beverage Intake	205	173 (84.4%)	32 (15.6%)
Vegetable Intake	204	168 (82.4%)	36 (17.7%)
Fruit Intake	205	169 (82.4%)	36 (17.6%)
Family Meals	204	168 (82.4%)	36 (17.7%)
Fast Food Intake	205	166 (81.0%)	39 (19.0%)
Breakfast	58	43 (74.1%)	15 (25.9%)
Dairy Intake	199	142 (71.4%)	57 (28.6%)

* Based on adult values for waist and hip circumferences. ** PSC, Pediatric Symptoms Checklist, clinically significant scores > 28.

**Table 3:** Changes in confidence, knowledge, likelihood, and influences of preparing whole food, plant-based meals in hospital employees.

Survey Question	Percentage of Participants		
How confident are you in your ability to:	Somewhat/Very Confident Pre (%)	Somewhat/Very Confident Post (%)	Change (%)
Incorporate seasonal foods into meals?	51.9	90.4	+38.5
Design and prepare a balanced plant-based meal?	50.0	88.5	+38.5
Identify foods growing in the seasonal garden?	48.8	84.6	+35.8
Identify a whole grain vs. a refined grain?	51.3	84.6	+33.3
Choose foods that are nutritious?	82.1	98.1	+16.0
Identify the difference between processed and unprocessed foods?	78.4	94.2	+15.8
Utilizing kitchen tools while preparing a meal?	84.0	96.2	+12.2
Use a kitchen knife while preparing a meal?	84.6	94.2	+9.6
How likely are you to:	Often/Always Pre (%)	Often/Always Post (%)	Change (%)
Eat plant-based meals cooked at home?	30.1	50.0	+19.9
Share your skills and/or knowledge about cooking?	40.4	57.1	+16.7
Prepare a new recipe?	57.8	68.9	+11.1
Engage in conversations about your food choices?	58.0	67.9	+9.9
How do the following values influence your food choices?	Somewhat/Very Important Pre (%)	Somewhat/Very Important Post (%)	Change (%)
Seasonal	50.6	80.8	+30.2
Social impact	34.8	63.5	+28.7
Environmental impact	51.3	76.9	+25.6
Grown locally	56.0	80.8	+24.8
Calorie density	70.4	86.3	+15.9
Cost	87.4	94.2	+6.8
Nutritional value	91.4	94.2	+2.8
Taste	97.5	100.0	+2.5

## Data Availability

Not applicable.
